# Standardized residency training in China: the new internal medicine curriculum

**DOI:** 10.1007/s40037-017-0378-5

**Published:** 2017-11-02

**Authors:** Jonathan Lio, Yanqing Ye, Hongmei Dong, Shalini Reddy, John McConville, Renslow Sherer

**Affiliations:** 1 Department of Internal Medicine, University of Chicago Medical Center, Chicago, IL USA; 2 Department of Medicine, Zhongnan Hospital, Wuhan University, Wuhan, China

**Keywords:** Medical education-graduate, Medical education-policy/administration, International health

## Abstract

China formally established a system of national standardized medical residency training in 2014, which affects the health of its 1.4 billion people. Accompanying this system were new guidelines and standards for internal medicine residency training. However, the majority of the standards focused on process measurements, such as minimum case requirements of diseases and procedural skills, rather than describing broader physician competencies in the domains of professionalism, patient care, communication, teamwork, quality improvement, and scholarship. While China has taken a large step forward with standardization of certain aspects of internal medicine residency training, the next step should focus on outcome measures and creating a system that is competency-based.

**Electronic supplementary material:**

The online version of this article (10.1007/s40037-017-0378-5) contains supplementary material, which is available to authorized users.

The year 2014 marked the formal establishment of a national standardized residency training system in mainland China, home to a population of 1.4 billion people. This was an effort led by the National Health and Family Planning Commission (NHFPC) to improve the quality of physician training. NHFPC commissioned the China Medical Doctor Association, a representative group of Chinese physicians under direct governance of the NHFPC, to create training content and accreditation standards. Residency programs were set at 3 years in duration regardless of specialty and had to comply with these standards. Thirty provinces (all provinces in China except Tibet) were carrying out resident training—55,000 resident physicians had enrolled in 8,500 residency programs housed in 559 hospitals—by the end of 2014 [1]. The government stated that by 2020, all new medical graduates looking for work in a clinical capacity must first complete residency training in an accredited program [2].

The training of China’s future healthcare practitioners deserves careful scrutiny and ongoing dialogue given its recent implementation and impact on one fifth of the world’s population. We draw on our experiences as clinician-educators working as part of a Sino-American joint university collaboration tasked with medical education reform to introduce the readership specifically to internal medicine residency training in China, first by describing the national internal medicine residency training guidelines and then analyzing them from the combined perspectives of Chinese and American medical educators.

## New standards for internal medicine residency training

The government released new standards for the internal medicine curriculum in 2014 [3]. Prior to this, each province conducted its training according to its own standards. However, 17 of the 18 pages of the new guidelines focused on lists of diseases and procedural skills, rather than describing standards of competencies in the broader domains of professionalism, communication, teamwork, and other key aspects.

Residents were required to finish 33 months of training in different departments within internal medicine to complete training. Twenty-nine of the 33 months were core rotations assigned in subspecialties (Table 1); none were allocated to general internal medicine wards. The remaining 4 months were electives, where residents could choose from core rotations or others such as geriatrics, oncology, radiology, or rural practice. Only 2 months were allotted for outpatient rotations.

**Table 1:** National rotation requirements for internal medicine residency

Rotation	Required time (months)
Cardiology	4
Pulmonology	3
Gastroenterology	3
Haematology	2
Nephrology	2
Rheumatology	2
Infectious diseases	2
Neurology	1.5
Psychology counselling clinic	0.5
Emergency medicine	3
Critical care medicine	2
Endocrinology	2
Outpatient	2
Elective	4
Total	33

The government-issued curriculum document provided guidelines for each subspecialty rotation divided into three sections: ‘rotation purpose’, ‘basic requirements’, and ‘advanced requirements’ (see online Electronic Supplementary Material). The rotation purpose section listed topics to understand or master, such as arrhythmia mechanisms, and classification and rational use of drugs commonly used in cardiovascular disease. Basic requirements were composed of lists of diseases and skills and the number of cases needed to fulfil the requirements of the rotation, such as five cases of congestive heart failure. Diseases and skills in the advanced requirements section, e. g. infectious endocarditis, did not have a minimum case requirement. The curriculum was designed in such a way that each subspecialty remained isolated from the others, as the emphasis for administrators was on fulfilling checklists rather than nurturing the growth of cross-departmental competencies.

Guidelines stated that assessments of residents should be conducted monthly, at the end of each rotation, and on a yearly basis. To be eligible to take the certification exam, residents must have successfully completed their rotations and met minimum requirements for numbers of cases and procedures.

Currently, outcomes monitored by the government include the number of residents trained in a standardized fashion and the passing rate of the residency certification exam. It should be noted, however, that the certification examination passing rate is not a stable outcome since the exam’s format has been changing; for example multiple choice questions were added in 2017 [4].

## Increasing focus on competencies

The prescribed guidelines from the national government described above represent a substantial step in standardizing residency training and serve as a foundation for improving the quality of resident graduates. However, the guidelines as currently formulated focus strongly on process measures. While process is essential, the logical next step is to focus on outcomes and competency [5], in alignment with the global medical education community, moving from a Flexner-type era to a competency-based era [6]. For example, in addition to requiring residents to see a minimum number of congestive heart failure cases, guidelines should also describe abilities regarding management of heart failure that the resident must attain by the end of residency.

To facilitate a clearer understanding of a physician’s competencies for medical educators and residents alike, a competency framework may offer a more interpretable means to describe what these abilities are, similar to what other countries such as Canada and the USA have done [7, 8]. Other medical educators have pointed out the lack of emphasis on subjects such as ethics and communication in the curriculum [9, 10]. With the commodification of healthcare impacting the patient-physician relationship and increased rates of violence against physicians [11], creating a framework focused on addressing issues such as communication may help alleviate some of these problems [12].

There also needs to be consensus on the endpoint of residency, or the minimal level of ability necessary to graduate from residency [13], as well as other milestones for the various competencies. Since many competencies may cross cultures [14], NHFPC could adapt an existing framework such as that of the Accreditation Council for Graduate Medical Education [15, 16]; however, researchers from China Medical University have already developed a competency framework that is consistent with the Chinese medical context [17].

A competency framework would guide future revisions of the internal medicine curriculum and standards regarding residency training. If the goal is to train independent practitioners, then resident responsibilities should be specified to reflect that goal. Likewise, the government can monitor outcomes based on resident competency as defined by the framework and assure the public of the results of training as the USA has done [18].

To address the ‘siloed’ nature of training in subspecialty departments, perhaps future guidelines can include required rotations in geriatrics or general wards, as Peking Union Medical College Hospital has done [19]. If NHPFC were to stimulate the creation or expansion of such wards for the purpose of teaching residents and for developing skills in cross-cutting competencies, as in geriatrics and general internal medicine, via incentives for teaching hospitals, both national goals of standardized internal medicine residency training programs and expanded primary care and general practice capacity would be well served. Furthermore, a curriculum with cross-departmental competencies would help unify the disparate subspecialty rotations into a whole, and would allow hospitals to better coordinate and consolidate training in disparate specialty wards and departments [20, 21].

Outpatient training is an area which is conspicuously sparse in the national curriculum guidelines. For internal medicine residents, out of the 3 years of rotations, only 2 months are required in clinic. For general practice residents, only 6 months are allocated to general practice clinic. In the USA, internal medicine residents must spend at least 1 year in the ambulatory setting [22]. This hospital orientation of training is not unique to China but is a global issue as well [20]. Although the clinic environment in China is high-throughput and does not allow easy integration of residents, in order to adequately prepare residents for future practice in which the clinic is a key component, there needs to be more focus on outpatient training and innovation [23, 24]. Residency training programs such as Sir Run Run Shaw Hospital’s [25] may provide some insight into how to better structure outpatient experiences for resident learning in the Chinese context.

## Conclusion

China’s postgraduate medical education has taken a large step forward with standardization of residency training, and its changes will affect the health of a fifth of the world’s population. We argue that while it has created a national curriculum detailing many process measures, the next step forward should focus on outcome measures and creating a system that is competency-based.

## Caption Electronic Supplementary Material

National standardized residency training content and standards for cardiology
