# Supplementary material for: Standardized residency training in China: the new internal medicine curriculum
Source: Perspect Med Educ. 2017 Nov 2;7(1):50–3. doi: 10.1007/s40037-017-0378-5 (PMC5807259; doi:10.1007/s40037-017-0378-5)
Supplement: Supplementary file 1 — National standardized residency training content and standards for cardiology [file 40037_2017_378_MOESM1_ESM.docx]

Appendix

National standardized residency training content and standards for cardiology (translated from Chinese)

**Department of Cardiology (4 months required rotation)**

1. Rotation Purpose

Mastery: anatomy and physiology of the cardiovascular system; anatomical and functional characteristics of cardiac conduction system; arrhythmia mechanisms and classification; pathogenesis, clinical manifestations, diagnosis, differential diagnosis, and treatment of common cardiovascular diseases; acute cardiovascular disease, diagnosis and treatment; rational use of drugs commonly used in cardiovascular disease; X-ray findings of common cardiovascular diseases; diagnosis of common ECG findings; electrical cardioversion technique.

Understand: basic knowledge of cardiac electrophysiology, pericardiocentesis, cardiac pacing, Holter monitoring, ambulatory blood pressure, echocardiography.

2. Basic Requirements

A. Diseases and case number requirements

| Disease | Minimum number |
| --- | --- |
| CHF | 5 |
| Common arrhythmias | 10 |
| Hypertension | 10 |
| Valvular heart disease | 3 |
| Myocarditis and cardiomyopathy | 2 |
| Coronary heart disease, angina | 8 |
| Dyslipidemia | 5 |
| Acute myocardial infarction | 5 |
| Diagnosis and management of common acute cardiovascular disease | 6 |

Required inpatient management of not less than 50 patients of which at least 25 patients were followed from admission to discharge.

B. Basic skills requirements

| Procedural skill name | Minimum number |
| --- | --- |
| X-ray diagnosis of common heart diseases | 20 (can describe accurately) |
| Electrical cardioversion | 2 |
| Perform 12-lead ECG and interpret common ECG patterns, including: left ventricular hypertrophy, left atrial hypertrophy, left bundle branch block, myocardial infarction, hypokalemia, hyperkalemia, sinus arrhythmia, WPW syndrome, escape rhythm, atrioventricular block, premature contraction, paroxysmal supraventricular tachycardia, atrial fibrillation, atrial flutter, ventricular tachycardia, ventricular fibrillation | 50 (independently writes report) |

3. Advanced Requirements

In addition to the basic requirements, the following diseases and skills should be studied.

A. Diseases

     Cardiac tamponade, pericardial disease, infective endocarditis, pulmonary vessel disease, common adult congenital heart disease, aortic disease.

B. Clinical knowledge, skill requirements

      Pericardiocentesis (understand), Holter monitor (participation), temporary and permanent cardiac pacing (understand), percutaneous coronary intervention (understand) ambulatory blood pressure (participation), common echocardiography (understand) cardiac radionuclide examination (understand).

C. Foreign language, teaching, research and other capabilities: Relevant literature review or a book report.
